# Prevalence, clinical characteristics, and predictors of peripheral arterial disease in hemodialysis patients: a cross-sectional study

**DOI:** 10.1186/s12882-019-1468-x

**Published:** 2019-07-26

**Authors:** Radislav R. Ašćerić, Nada B. Dimković, Goran Ž. Trajković, Biljana S. Ristić, Aleksandar N. Janković, Petar S. Durić, Nenad S. Ilijevski

**Affiliations:** 1 Department of Vascular Surgery Clinic of Surgery, Clinical Hospital Center Zvezdara, Dimitrija Tucovića 161, Belgrade, 11000 Serbia; 2 Clinic of Nephrology, Clinical Hospital Center Zvezdara, Belgrade, Serbia; 3 Medical Faculty, University of Belgrade, Belgrade, Serbia; 4 Department of Medical Statistics and Informatics Medical Faculty, University of Belgrade, Belgrade, Serbia; 5 Primary Health Center, Palilula, Belgrade, Serbia; 6 Vascular Surgery Clinic, Dedinje Cardiovascular Institute, Belgrade, Serbia

**Keywords:** Peripheral arterial disease, Ankle-brachial index, Hemodialysis, C-reactive protein, Hickman vascular access

## Abstract

**Background:**

Peripheral arterial disease (PAD) is common in patients with end-stage renal disease on hemodialysis, but is frequently underdiagnosed. The risk factors for PAD are well known within the general population, but they differ somewhat in hemodialysis patients. This study aimed to determine the prevalence of PAD and its risk factors in patients on hemodialysis.

**Methods:**

This cross-sectional study included 156 hemodialysis patients. Comorbidities and laboratory parameters were analyzed. Following clinical examinations, the ankle-brachial index was measured in all patients. PAD was diagnosed based on the clinical findings, ankle-brachial index < 0.9, and PAD symptoms.

**Results:**

PAD was present in 55 of 156 (35.3%; 95% CI, 27.7–42.8%) patients. The patients with PAD were significantly older (67 ± 10 years vs. 62 ± 11 years, *p* = 0.014), more likely to have diabetes mellitus (*p* = 0.022), and anemia (*p* = 0.042), and had significantly lower serum albumin (*p* = 0.005), total cholesterol (*p* = 0.024), and iron (*p* = 0.004) levels, higher glucose (*p* = 0.002) and C-reactive protein (*p* < 0.001) levels, and lower dialysis adequacies (*p* = 0.040) than the patients without PAD. Multivariate analysis showed higher C-reactive protein level (odds ratio [OR], 1.03; 95% confidence interval [CI], 1.00–1.06; *p* = 0.030), vascular access by Hickman catheter (OR, 4.66; 95% CI, 1.03–21.0; *p* = 0.045), and symptoms of PAD (OR, 5.20; 95% CI, 2.60–10.4; *p* < 0.001) as independent factors associated with PAD in hemodialysis patients.

**Conclusion:**

The prevalence of PAD was high among patients with end-stage renal disease on hemodialysis. Symptoms of PAD, higher C-reactive protein levels, and Hickman vascular access were independent predictors of PAD in patients on hemodialysis.

## Introduction

Peripheral arterial disease (PAD) of the lower extremities is widespread, and it affects over 200 million people worldwide [1]. PAD is also highly prevalent among patients with end-stage renal disease, and it has serious consequences that influence patient outcomes [2, 3]. The prevalence of PAD has increased over the last decade, particularly in low-income countries [4]; further, it increases with age and is 4.5–14.5% more prevalent among individuals aged ≥65 years [5]. Among patients on hemodialysis (HD), the prevalence of PAD is much higher, ranging from 17 to 48% [6, 7].

The findings from the Dialysis Outcomes and Practice Patterns Study (DOPPS) described a PAD prevalence of 25.3% among patients on HD that was accompanied by significant geographic variations. In European countries, PAD prevalence was 17.5–37.8%, while in Japan¸ the prevalence was significantly lower at 11.5% [8]. Similarly, the findings from the Hemodialysis Study (HEMO) showed that PAD was seen in 23% of patients on HD [9]. The findings from the National Health and Nutrition Examination Survey (NHANES) 1999–2000 showed that PAD was present in 24% of patients with renal insufficiency and in 3.7% of participants with normal renal function; these findings were independent of diabetes, age, and hypertension [10]. Japanese investigators have shown that PAD prevalence in patients on HD ranges from 23.8 to 47%, depending on the study population [2, 11]. Although these studies were valuable, they had different methods for PAD diagnosis.

The early diagnosis and management of PAD can improve the prognosis for patients on HD [11]. Therefore, many patients on HD with PAD could avoid or at least delay adverse events, such as amputations, cardiovascular events, and death if PAD was timely diagnosed and adequately treated [12]. The traditional risk factors for PAD, namely, age, diabetes mellitus (DM), smoking, hyperlipidemia, hypertension, and male sex, are well recognized within the general population [7], but these risk factors are supplemented by other risk factors in patients on HD [13, 14]. To our knowledge, no studies have previously investigated the effects of vascular access on PAD prevalence. Thus, this study aimed to examine the prevalence of PAD, the risk factors associated with PAD, and the clinical characteristics of PAD in patients on HD.

## Methods

### Study population

The study subjects comprised 197 outpatients who were on maintenance HD at the Clinical Department for Nephrology and Metabolic Disorders with Dialysis at Zvezdara University Medical Center. Of these subjects, 41 patients were excluded from the study because 36 did not provide informed consent and five died during the study and their data were incomplete. Hence, 156 patients participated in this cross-sectional study between January 2016 and January 2017. Zvezdara University Medical Center’s ethics committee approved this study, and all participants provided written informed consent before study initiation.

### Risk factors and comorbid diseases

The patients’ medical records were reviewed, and data describing their demographic and clinical characteristics were collected, including age, sex, height, weight, HD duration and type, and causes of renal failure. The following risk factors and comorbidities associated with PAD were analyzed: smoking habits, hypertension, hyperlipidemia, DM, anemia, coronary artery disease, and cerebrovascular diseases, including stroke. The body mass index was calculated as the weight in kilograms divided by height in meters squared.

### Laboratory parameters

Blood samples were taken quarterly at the beginning of the second HD session of the week. The analyzed laboratory parameters included the serum creatinine, urea, potassium, calcium, albumin, phosphorus, total cholesterol, triglyceride, glucose, C-reactive protein (CRP), iron, transferrin saturation, ferritin, intact parathyroid hormone, dialysis adequacy (Kt/V), hemoglobin levels and blood cell counts. Mean values of four measurements were used in the analyses.

### Patient assessments and clinical examinations

Blood pressure measurements were obtained before and after dialysis and during three consecutive dialysis sessions, and the mean values were used to calculate the pulse pressure (systolic pressure – diastolic pressure) and the mean arterial pressure (diastolic pressure + 1/3 pulse pressure). The presence of left ventricular hypertrophy was determined indirectly using electrocardiography.

The patients’ histories of previous peripheral artery interventions, including bypass surgery, percutaneous interventions, or amputations for PAD, were documented. The patients were asked about PAD symptoms, including claudication, coldness, numbness, and resting pain, and they were categorized using the Fontaine classification of PAD symptoms [15].

All patients underwent standard physical examinations that included pulse palpation on the femoral artery, popliteal artery, dorsalis pedis artery, and posterior tibial artery on both legs, and auscultation for femoral bruits. The pulses were coded as absent (0), weak (1), or present (2). To ensure that the findings were comparable, all patients were examined by the same experienced physician.

### Ankle-brachial index (ABI) measurements

We measured ABI using a portable handheld bidirectional Doppler device (Life Dop 250 ABI; Wallach Surgical Devices, Trumbull, CT, USA). All ABIs were measured at room temperature and before dialysis while the patients were unclothed, in a supine position, and after they had rested for 5–10 min. The systolic blood pressure was measured in the brachial artery of the arm without vascular access and in the dorsalis pedis artery and posterior tibial artery of the right and left lower limbs after using a Doppler probe of appropriate cuff size. The same well-trained nurse performed all ABI measurements. The ABI was calculated by dividing the ankle systolic pressure by arm systolic pressure. The lower ABI value of both legs was used as the leg index. An ABI was classified as low (< 0.9) if any of the ABI values were < 0.9, normal (0.9–1.3) if all four values were between 0.9 and 1.3, and high (> 1.3) if at least one ABI was > 1.3 with other values being normal [16].

Patients were diagnosed with PAD if they fulfilled the criteria relating to clinical examinations, Fontaine classification, and ABIs. The patients were considered to have PAD if they had an ABI < 0.9 or if they had claudication and absence of pulses, despite having normal ABIs. We performed duplex ultrasonography on patients who had normal and high ABIs, and confirmed PAD if there was ≥50% narrowing of the large lower extremity blood vessels that had caused either sub-occlusion or occlusion of ≥1 crural arteries.

### Statistical analyses

The R software environment for statistical computing [17] was used to conduct the statistical analyses. The significance level was set at 0.05. Continuous variables are presented as the means and standard deviations, and categorical variables are presented as numbers and percentages; these descriptive statistics were used to characterize the study sample. The differences between groups in relation to the continuous variables were analyzed using Student’s t-test and the Mann–Whitney U test for the independent samples. Pearson’s chi-squared test or Fisher’s exact test were used to analyze the differences between groups in relation to the categorical variables. A multivariate logistic regression model was constructed to determine the predictors of PAD, which included the statistically significant variables (*P* < 0.05) from the univariate analyses.

## Results

There were no statistically significant differences between patients who did and did not participate in the study in terms of age, sex, and HD vintage. Of the 156 patients identified, PAD was present in 55 (35.3%; 95% CI, 27.7–42.8%), of which 40% were women (Table 1). Hypertension was the most common condition that caused the end-stage renal disease (53.2%), and DM was the second most common cause of the end-stage renal disease (12.8%) in our patients. Compared with the non-PAD group, the PAD patients were older, more likely to be male, and had higher incidence rates of anemia and DM. The participants’ mean HD vintage was 71.7 ± 69.5 (median: 55; range: 0–354) months. The groups did not differ in relation to the HD type and daily HD duration. Type of vascular access significantly differed between the PAD and non- PAD groups (*p* = 0.004). The prevalence of PAD was the highest in patients with Hickman vascular catheter (75%), followed by patients with arteriovenous graft (AVG; 53.8%), and was the lowest in patients with arteriovenous fistula (AVF; 30%). The only statistically significant difference in PAD prevalence between these groups was found when patients with Hickman vascular catheter and AVF were compared (*p* = 0.003). Although the prevalence of PAD was greater in patients with AVG than in those with AVF, there was no significant difference (*p* = 0.117).

**Table 1:** Demographic, baseline and dialysis characteristics of the study population

Characteristics	Total	PAD	No PAD	*p*
	156 (100)	55 (35.3)	101 (64.7)	
Age years mean ± SD	64 ± 11	67 ± 10	62 ± 11	0.014
Gender, n (%)				0.905
Male, n (%)	96 (61.5)	33 (60.0)	63 (62.4)	
Primary kidney disease, n (%)				0.118
Hypertension	83 (53.2)	28 (50.9)	55 (54.5)	
Diabetes mellitus	20 (12.8)	12 (21.8)	8 (7.9)	
Glomerulonephritis	18 (11.5)	4 (7.3)	14 (13.9)	
Cystic disease	12 (7.7)	2 (3.6)	10 (9.9)	
Pyelonephritis and Obstructive uropathy	10 (6.4)	4 (7.3)	6 (5.9)	
Other	13 (8.3)	5 (9.1)	8 (7.9)	
BMI, mean ± SD kg/m2	24.0 ± 4.1	24.2 ± 4.2	23.9 ± 4.0	0.68
Hypertension, n (%)	142 (91.0)	53 (96.4)	89 (88.1)	0.14
Hyperlipoproteinemia, n (%)	75 (48.1)	22 (40.0)	53 (52.5)	0.136
Diabetes mellitus, n (%)				0.022
No	117 (75.0)	34 (61.8)	83 (82.2)	
Type II oral therapy	16 (10.3)	8 (14.5)	8 (7.9)	
Type II Insulin	20 (12.8)	12 (21.8)	8 (7.9)	
Type I Insulin	3 (1.9)	1 (1.8)	2 (2.0)	
CAD, n (%)	54 (34.6)	20 (36.4)	34 (33.7)	0.801
Angina pectoris, n (%)	45 (28.8)	15 (27.3)	30 (29.7)	0.750
Myocardial infarction, n (%)	14 (9.0)	8 (14.8)	6 (5.9)	0.081
CABG, PCI, n (%)	15 (9.6)	8 (14.8)	7 (6.9)	0.153
Stroke n (%)	6 (3.8)	4 (7.3)	2 (2.0)	0.186
Anemia, n (%)	144 (92.3)	54 (98.2)	90 (89.1)	0.042
Smoking, n (%)				0.677
No	82 (52.6)	27 (49.1)	55 (54.5)	
Yes	39 (25.0)	16 (29.1)	23 (22.8)	
Past	35 (22.4)	12 (21.8)	23 (22.8)	
LVH, n (%)	17 (10.9)	5 (9.8)	12 (12.9)	0.582
Access for HD, n (%)				0.004
AVF	130 (83.3)	39 (70.9)	91 (90.1)	
CVC	1 (0.6)	0 (0.0)	1 (1.0)	
Hickman vascular catheter	12 (7.7)	9 (16.4)	3 (3.0)	
AVG	13 (8.3)	7 (12.7)	6 (5.9)	
HD vintage (months), median (range)	55 (0, 354)	55 (0, 264)	55 (0, 354)	0.867
Type of HD, n (%)				0.086
HDF	29 (18.6)	6 (10.9)	23 (22.8)	
High flux HD	127 (81.4)	49 (89.1)	78 (77.2)	
Kt/V median (range)	1.34 (0.51, 3.35)	1.31 (0.82, 2.14)	1.37 (0.51, 3.34)	0.040

*AVF* Arteriovenous fistula, *AVG* Arteriovenous graft, *BMI* Body mass index, *CAD* Coronary artery disease, *CABG* coronary artery bypass graft, *CVC* Central venous catheter, *HD* Hemodialysis, *HDF* Hemodiafiltration, *LVH* Left ventricular hypertrophy, *PAD* Peripheral arterial disease, *PCI* Percutaneous coronary intervention

The PAD group had a lower median Kt/V value than the non-PAD group (1.31 [range: 0.82–2.14] vs. 1.37 [range: 0.51–3.34]; *p* = 0.04). Two patients had undergone major amputations as a consequence of PAD; specifically, one had above-the-knee amputation and one had below-the-knee amputation. Four patients had undergone minor amputations, comprising two who had amputations of one toe and two who had amputations of a little toe. One patient had undergone an abdominal aortic aneurysm resection and another patient had undergone a femoro-femoral cross-over bypass. The patients in the PAD and non-PAD groups did not differ in relation to the systolic blood pressure, diastolic blood pressure, pulse pressure, and mean arterial pressure values determined before and after HD.

**Fig. 1:**
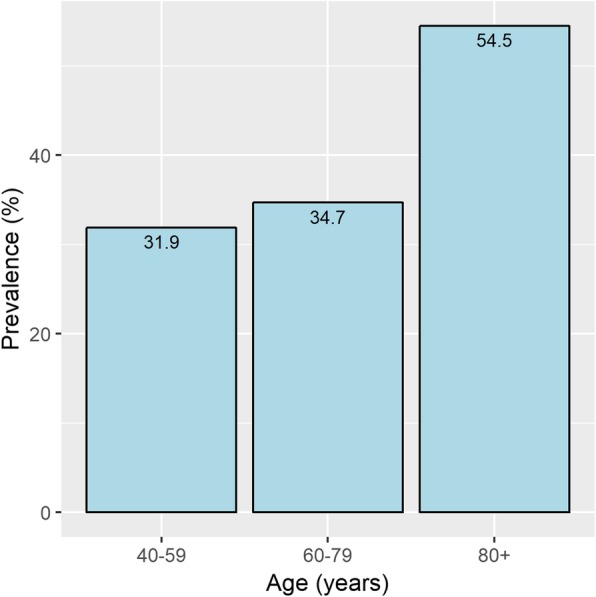
Prevalence of peripheral arterial disease in the hemodialysis patients according to age. X title: Age (years). Y title: Prevalence (%)

The prevalence of PAD was associated with the age of patients on HD, and it was most prevalent among patients who were ≥ 80 years old (Fig. 1).

Table 2 presents the participants’ symptoms and clinical findings. Of the patients on HD without PAD, 19.8% had various symptoms in their legs, including coldness and numbness, and 2% had claudications. The PAD and non-PAD groups differed significantly in relation to the clinical findings, including pulse palpation, femoral bruit auscultation, and ABI values (all *p* < 0.001).

**Table 2:** Fontaine symptoms and the study participants’ clinical findings

	Total	PAD	No PAD	*P*
PAD Symptoms Fontaine, n (%)				
I	96 (61.5)	15 (27.3)	81 (80.2)	<0.001
IIa	37 (23.7)	19 (34.5)	18 (17.8)	
IIb	21 (13.5)	19 (34.5)	2 (2.0)	
III	1 (0.6)	1 (1.8)	0 (0.0)	
IV	1 (0.6)	1 (1.8)	0 (0.0)	
Femoral bruit, n (%)				
Yes	45 (28.9)	28 (50.9)	17 (16.8)	<0.001
No	106 (67.9)	23 (41.8)	83 (82.2)	
Pulse, n (%)				
Femoral				
No	1 (0.6)	1 (1.8)	0 (0)	0.042
Weak	2 (1.3)	2 (3.6)	0 (0)	
Yes	153 (98.1)	52 (94.5)	101 (100)	
Popliteal				
No	22 (14.1)	22 (40.0)	0 (0)	<0.001
Weak	8 (5.1)	7 (12.7)	1 (1)	
Yes	126 (80.8)	26 (47.3)	100 (99)	
DPA				
No	46 (29.5)	37 (67.3)	9 (8.9)	<0.001
Weak	31 (19.9)	9 (16.4)	22 (21.8)	
Yes	79 (50.6)	9 (16.4)	70 (69.3)	
PTA				
No	50 (32.1)	41 (74.5)	9 (8.9)	<0.001
Weak	39 (25.0)	11 (20.0)	28 (27.7)	
Yes	67 (42.9)	3 (5.5)	64 (63.4)	
ABI indexes n (%)				
ABI < 0.9	41 (26.3)	41 (74.5)	0 (0.0)	<0.001
ABI > 1.3	36 (23.1)	7 (12.7)	29 (28.7)	
ABI 0.91–1.3	79 (50.6)	7 (12.7)	72 71.3)	

*ABI* Ankle-brachial index, *DPA* Dorsalis pedis artery, *PAD* Peripheral arterial disease, *TPA* Tibialis posterior artery

**Fig. 2:**
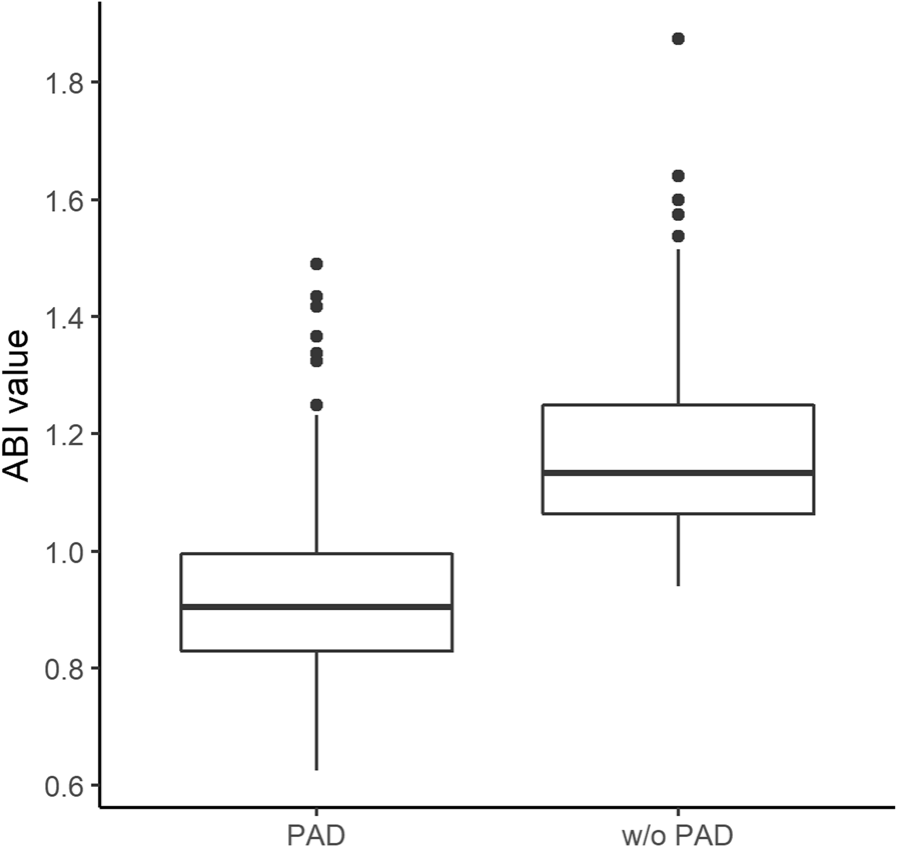
The median ankle-brachial index values in the hemodialysis patients with and without peripheral arterial disease. X title: PAD, w/o PAD. Y title: ABI value

The patients on HD with PAD had significantly lower median ABIs than the patients on HD without PAD (0.90 vs. 1.13; *p* < 0.001) (Fig. 2).

Table 3 summarizes the laboratory blood test results. The albumin, total cholesterol, and iron levels were lower and the glucose and CRP levels were higher in the PAD group than in the non-PAD group.

**Table 3:** Biochemical characteristics of the study population

	Total	PAD	No PAD	*P*
Urea, mmol/l	23.1 ± 4.7	23.3 ± 5.1	22.9 ± 4.4	0.663
Creatinine, μmol/l	840 ± 164	837 ± 182	841 ± 155	0.878
Albumin, g/L	37 ± 2	36 ± 2	38 ± 2	0.005
Glucose, mmol/l	6.4 ± 1.9	7.0 ± 2.1	6.0 ± 1.7	0.002
Total cholesterol, mmol/l	4.2 ± 1.0	4.0 ± 0.8	4.4 ± 1.1	0.024
Triglyceride, mmol/l	1.83 ± 0.93	1.82 ± 1.07	1.83 ± 0.85	0.934
Erythrocytes, × 10^12^/L	3.42 ± 0.30	3.41 ± 0.29	3.43 ± 0.30	0.663
Hemoglobin, g/dL	10.4 ± 0.9	10.3 ± 0.8	10.4 ± 1.0	0.373
Iron, μmol/l mean ± SD	10.9 ± 3.3	9.9 ± 3.0	11.5 ± 3.3	0.004
T-Sat.,% median (range)	0.29 (0.08–7.49)	0.28 (0.08–0.54)	0.29 (0.15–7.49)	0.064
Ferritin, ng/mL median (range)	279 (28–1126)	285 (44–906)	274 (28–1126)	0.201
White blood cell, ×10^9^/L	7.05 ± 2.25	7.47 ± 2.19	6.82 ± 2.26	0.086
CRP, mg/L median (range)	7.3 (0.4–104.3)	9.7 (0.9–104.3)	6.6 (0.4–64.9)	< 0.001
K, mean ± SD	5.22 ± 0.98	5.20 ± 1.40	5.24 ± 0.65	0.775
Calcium, mmol/l	2.27 ± 0.17	2.26 ± 0.15	2.27 ± 0.17	0.732
Phosphorus, mmol/l	1.50 ± 0.42	1.51 ± 0.43	1.48 ± 0.41	0.74
Parathormone, pg/ml, median (range)	131 (6.1–4184)	123 (14.6–1564.6)	140 (6.1–4184.0)	0.292

*CRP* C-reactive protein, *PAD* Peripheral arterial disease, *T-sat* Transferrin saturation

Table 4 presents the results of logistic regression analyses using PAD as the dependent variable. The univariate analysis indicated that age, DM, symptoms based on the Fontaine classification, vascular access provided by a Hickman catheter, lower albumin and total cholesterol levels, and a higher CRP level were associated with PAD (all *p* < 0.05). The multivariate logistic regression analysis showed that the independent factors associated with PAD in patients on HD were a higher CRP level (odds ratio [OR], 1.03; 95% confidence interval [CI], 1.00–1.06; *p* = 0.030), vascular access provided by a Hickman catheter (OR, 4.66; 95% CI, 1.03–21.0; *p* = 0.045), and presence of PAD symptoms based on the Fontaine classification (OR, 5.20; 95% CI, 2.60–10.4; *p* < 0.001).

**Table 4:** Logistic regression analyses using peripheral arterial disease as the dependent variable

	Univariate model		Multivariate model	
Independent variable	OR (95% CI)	*P*	OR (95% CI)	*P*
Age	1.04 (1.01–1.07)	0.004	1.01 (0.97–1.06)	0.536
Diabetes mellitus	2.85 (1.35–6.00)	<0.001	1.29 (0.48–3.51)	0.817
Anemia	0.15 (0.02–1.21)	0.074	0.31 (0.03–3.00)	0.313
PAD symptoms by Fontaine	6.55 (3.55–12.1)	<0.001	5.20 (2.60–10.4)	<0.001
Access Hickman	4.74 (1.39–16.2)	0.013	4.66 (1.03–21.0)	0.045
Kt/v	0.36 (0.13–1.06)	0.064	1.04 (0.27–4.07)	0.953
Albumin	0.84 (0.74–0.96)	0.008	1.04 (0.88–1.25)	0.627
Total cholesterol	0.64 (0.43–0.94)	0.023	0.83 (0.50–1.37)	0.462
CRP	1.04 (1.02–1.07)	0.002	1.03 (1.00–1.06)	0.030

*CRP* C-reactive protein, *PAD* Peripheral arterial disease

## Discussion

Approximately 35% of patients on HD in this study had PAD, which concurs with the rates published to date [6, 7]. In this study, PAD was diagnosed based on an ABI of < 0.9, clinical findings, and categorization of symptoms based on the Fontaine classification; 41 patients with PAD had ABI values < 0.9, seven patients had ABI values > 1.3, and seven had ABI values between 0.9 and 1.3, or more specifically, between 0.91 and 1.0. We confirmed PAD in seven patients with normal and in seven patients with high ABI values using color Doppler ultrasonography. There were significant differences between the PAD and non-PAD groups with respect to the clinical findings, including those from pulse palpation and femoral bruit auscultation, and ABIs. The clinical findings were noticeably worse in the PAD group than in the non-PAD group. Often, these patients have DM; chronic kidney disease alone leads to arterial incompatibility as a consequence of pronounced calcification, and arterial stiffness generates false-positive ABIs [2, 6, 16]. An ABI < 0.9 alone should not be used to assess the severity of PAD in patients on HD [2]. An ABI of < 0.9, in conjunction with the clinical findings, namely, absence of pulses and presence of femoral bruits, and PAD symptoms, including claudication, would provide a more robust foundation for a diagnosis of PAD.

Measuring the ABI is simple, reliable, inexpensive, and non-invasive [18]. According to the most recent American Heart Association guidelines, ABIs of 0.9–1.09 are lower normal values [18]; however, some Japanese authors have recommended ABIs of 1.05–1.1 as the limit for a diagnosis of PAD in patients on HD [2, 11].

Intermittent claudication is the classic and most striking symptom associated with PAD, and it is relatively rare in the general population and in patients on HD [5, 19]. Therefore, intermittent claudication alone is not a reliable criterion for a diagnosis of PAD [6]. The findings from preliminary studies have shown that 20–50% of patients with PAD who had ABIs of < 0.9 did not report claudication; hence, these patients belong to the asymptomatic group of patients with PAD [5, 20]. In the UK, Webb et al. [21] studied 325 patients receiving HD and reported that 19% of these patients had claudication. In this study, 37.2% of the patients had claudication. In the DOPPS and HEMO studies of patients on HD, a diagnosis of PAD was based on a previous PAD diagnosis, the presence of intermittent claudication or critical ischemia of the extremities, previous surgical revascularization or amputation as a consequence of PAD, and prior diagnosis of an abdominal aortic aneurysm. Based on these criteria, the rates of PAD in the DOPPS and HEMO studies were 25.3 and 23%, respectively [8, 12]. Likewise, the prevalence of PAD in the NHANES was 24% in the group of patients with chronic kidney disease, and the same criteria were used to diagnose PAD [10].

In studies that used ABI < 0.9 as a criterion for PAD diagnosis in patients on HD, PAD prevalence was slightly higher and it reached 37% [6]. Because ABIs < 0.9 are relevant for a diagnosis of PAD, they are an important prognostic factor associated with cardiovascular disease and mortality in patients on HD [11, 16, 19].

In our study, PAD was associated with older age, DM, and anemia, and a high percentage of patients with PAD had hypertension; however, the rate of left ventricular hypertrophy was not significantly higher in this group of patients, perhaps because it was determined by electrocardiography. Significant differences did not exist with respect to the systolic and diastolic blood pressure values determined before and after HD nor in relation to the pulse pressure and mean arterial pressure values in this study, but other investigators have described significantly higher diastolic and pulse pressure values in patients with PAD on HD [12, 13]. Viazzi et al. [3] suggested that the predictive power associated with ambulatory interdialytic blood pressure monitoring was much greater than the predictive powers of blood pressure measurements attained in the clinic or those determined immediately before and after dialysis. Our patients on HD were closely monitored and their blood pressure was well controlled, regardless of the presence of PAD.

Several authors have described higher frequencies of cardiovascular disease and stroke among patients with PAD [5, 22]. Although the patients in this study had high rates of cardiovascular disease and stroke, the groups did not differ with regard to their frequencies. Anemia was present among patients with PAD, a finding that concurs with the findings from a previously published study that showed that patients with PAD with anemia had a more severe form of the disease and had higher risks of mortality and limb amputation than patients with PAD without anemia [23].

Whereas the traditional risk factors associated with PAD, namely, age, DM, smoking status, hypertension, male sex, and hyperlipidemia [8], are valid within the general population, the risk factors for PAD slightly differ among patients on HD [6, 13]. In addition to the traditional risk factors, renal-specific factors are associated with PAD in patients on HD [6, 12, 13]. A smaller number of studies have investigated correlations between PAD and its risk factors in patients on HD [24]. The findings from the HEMO study that included 936 patients on HD, showed that DM, age, and smoking status are associated with PAD, while the other conventional risk factors, namely, male sex, hypertension, and hypercholesterolemia, are not associated with PAD [9].

The findings from the United States Renal Data System study and the Dialysis Morbidity and Mortality Study, which examined the associations among the variables related to dialysis and the conventional cardiovascular risk factors for PAD in patients on dialysis, showed that a diagnosis of PAD was associated with age, male sex, being Caucasian, DM, smoking status, higher diastolic blood pressure, and left ventricular hypertrophy. In an adjusted analysis, PAD was also associated with low serum parathyroid hormone levels, a longer dialysis vintage, a lower Kt/V, and a low serum albumin level [25]. Compared with the non-PAD group, the HD vintage was shorter in the PAD group in this study, but the difference was not significant, which concurs with the findings from a study carried out in Japan [12]; however, other investigators consider that PAD is related to the dialysis vintage [14]. Lower Kt/V values were observed among patients in the PAD group, which is consistent with the findings from other studies [11, 25]. These findings might help explain why some uremic toxins that are not removed by dialysis, for example, dimethylarginine, accumulates, damages endothelial cells, and causes atherogenesis [19].

Chronic uremia is associated with systemic inflammation that causes hypoalbuminemia and increases the risk of PAD [13, 26], and may play an important role in the pathogenesis and progression of atherosclerosis [13]. Chronic inflammation, malnutrition, and hyperphosphatemia are associated with PAD in patients with end-stage renal disease [6, 7]. The serum albumin and total cholesterol levels indicate not only a patient’s nutritional status but also chronic inflammation [25, 26]. Serum CRP is related not only to vascular inflammatory reactions but also promotes atherogenesis and atherothrombosis [27]. In this study, patients with PAD had significantly higher CRP levels and lower albumin, total cholesterol, and iron levels, which are indirect indicators of malnutrition, inflammation, and atherosclerosis syndrome [28].

Similar to our study, several investigators have shown that hyperlipidemia is not a risk factor for PAD in patients on HD [12, 29], but some authors have described hyperlipidemia as a risk factor for PAD [5, 11]. In contrast, the findings from one study indicated that hyperlipidemia had a protective effect against PAD [29].

The patients with PAD in our study had significantly higher levels of glycemia, which correlated with the higher incidence of diabetes in these patients. Several authors have described DM and poor glycemic control as important risk factors for PAD [5, 8, 13, 26]. Earlier reports have suggested that inflammation plays a key role in atherosclerosis and confirmed that patients with elevated CRP and lower albumin levels have high occurrence rates of PAD [27]. The findings from this study concur with those from previous studies.

A distal radiocephalic AVF is the vascular access of choice for HD at our center because it is associated with the longest survival times and the lowest number of complications. Patients with inadequate blood vessels undergo AVG implantations. Tunneled Hickman vascular catheters are used for permanent vascular access in patients who have poor prognoses and shorter life expectancies and whose blood vessels are inappropriate for an AVF or AVG implantations [30]. Our data showed that compared with the patients without Hickman vascular catheters (25%), a significantly higher number of patients on HD with Hickman vascular catheters had PAD (75%). We also observed that patients with PAD and Hickman vascular catheter had significantly lower hemoglobin (*p* = 0.001), and albumin (*p* = 0.022) levels than patients with AVF. Compared to the patients with AVF, patients with implanted Hickman vascular catheter and AVG had significantly higher CRP values (*p* = 0.05). The presence of a permanent vascular catheter was a predictor of PAD, and the use of the catheter may be associated with silent or overt inflammation. As is already known, there is a strong correlation between the atherosclerosis of blood vessels of the arm and PAD. Moreover, an increased incidence of vascular access failure was noted in patients with AVG compared to those with AVF [16, 31].

The management and treatment of patients with PAD requires a multidisciplinary approach [32]. The National Kidney Foundation’s Kidney Disease Outcomes Quality Initiative guidelines [33] recommend that at the beginning of a dialysis session, all patients should be screened for PAD, be clinically examined, and have their ABIs measured. These patients often have insufficient daily activity and diabetic neuropathy, and they are usually asymptomatic; due to this, they are underdiagnosed and undertreated [12, 20].

Once PAD is diagnosed, all risk factors must be rigorously managed, which includes smoking cessation, administering aspirin, statins, and cilostazol, and undertaking active exercise [19, 22]. Timely diagnosis of PAD and its early treatment can improve patients’ quality of life and avoid or, at least, delay undesirable events, including amputation or death [12, 13].

### Study limitations

This was a single-center study that involved a relatively small number of patients. Additionally, for the purposes of vascular access, ABI was calculated based on the systolic blood pressure that was measured on one side only, which may have affected the findings as a consequence of subclavian steal syndrome and underestimated the values in some patients. We evaluated left ventricular hypertrophy based on electrocardiography, and we did not perform echocardiography. Although we determined the total cholesterol and triglyceride values, there were no data that described the high- and low-density lipoprotein cholesterol levels. We performed a cross-sectional study of prevalent patients on HD; however, a long-term study that monitors the relationships between the risk factors and the development of PAD in incident patient’s, would generate more robust data.

## Conclusion

The prevalence of PAD was high in our group of patients on HD. Independent predictors of PAD were the presence of symptoms based on the Fontaine classification, a higher CRP level, and vascular access provided by a Hickman catheter for HD. An ABI < 0.9 combined with the clinical findings and claudication more accurately signifies clinically significant PAD. An ABI < 0.9 alone, without clinical findings, is not sufficient for the detection of clinically significant PAD in patients on HD. Adequate and timely diagnosis and treatment of PAD could improve the quality of life and postpone complications in these patients.

## Data Availability

The dataset used in a current study are available from the corresponding author on reasonable request.

## Abbreviations

ABI: Ankle-brachial index
AVF: Arteriovenous fistula
AVG: Arteriovenous graft
BMI: Body mass index
CABG: Coronary artery bypass graft
CAD: Coronary artery disease
CRP: C-reactive protein
CVC: Central venous catheter
DOPPS: Dialysis Outcomes and Practice Patterns Study
DPA: Dorsalis pedis artery
HD: Hemodialysis
HEMO: Hemodialysis Study
LVH: Left ventricular hypertrophy
NHANES: National Health and Nutrition Examination Survey
PAD: Peripheral arterial disease
PCI: Percutaneous coronary intervention
TPA: Tibialis posterior artery
